# A versatile plasmid platform for auxotrophic complementation in attenuated *Mycobacterium bovis* BCG

**DOI:** 10.1007/s11033-026-11830-x

**Published:** 2026-04-28

**Authors:** Andriele Bonemann Madruga, Jady Duarte Nogueira, Mara Andrade Colares Maia, Natasha Rodrigues de Oliveira, Maria Eduarda Ehlert, Valentina Gessinger Ferreira, Bruna Silveira Pacheco, Fabiana Kommling Seixas, Tiago Veiras Collares, Izani Bonel Acosta, Antônio Sergio Varela, Odir Antônio Dellagostin, Thaís Larré Oliveira Bohn

**Affiliations:** 1 Laboratório de Vacinologia, Centro de Desenvolvimento Tecnológico, Universidade Federal de Pelotas, Pelotas, Rio Grande do Sul Brasil; 2 Laboratório de Biotecnologia do Câncer, Centro de Desenvolvimento Tecnológico, Universidade Federal de Pelotas, Pelotas, Rio Grande do Sul Brasil; 3 Grupo de Pesquisa em Reprodução Animal Comparada, Faculdade de Medicina Veterinária, Universidade Federal de Pelotas, Pelotas, Rio Grande do Sul Brasil

**Keywords:** Biobrick™, Mycobacteria, pUP500, Recombinant vaccine, Antibiotic resistance

## Abstract

**Background:**

*Mycobacterium bovis* BCG is widely used as a vaccine vector due to its safety, genetic stability, and immunomodulatory properties. However, current expression systems for BCG transformation rely on antibiotic resistance genes, raising biosafety concerns. Therefore, alternative strategies—such as the use of auxotrophic strains—are highly desirable. Our group previously employed BioBrick™ technology to construct the pUP500 plasmid series containing various mycobacterial promoters. We engineered these plasmids for the auxotrophic complementation of BCG ΔLeuD, a leucine-auxotrophic strain, using an antibiotic-free and BioBrick™-compatible approach.

**Methods and Results:**

The auxotrophic complementation cassette pAN-*leuD* was cloned into pUP500 plasmids. Subsequently, the *egfp* gene was inserted downstream of the promoters, the kanamycin resistance gene was removed, and fluorescence was assessed by flow cytometry, qPCR and confocal microscopy in BCG grown in 7H9 medium and inside macrophages. Auxotrophic complementation was confirmed by growth of transformants in non-selective medium and analysis of *leuD* expression, and plasmid stability was evaluated along six BCG generations. Strains harboring plasmids containing the pAN and HspX promoters exhibited stronger fluorescence levels both *in vitro* and within macrophages. In contrast, the 18 kDa promoter demonstrated marked activation specifically in the intracellular environment, driving robust gene expression inside macrophages. These expression patterns were significantly higher compared to strains carrying the Ag85B or Hsp60 promoters, as well as to the negative control. Except for the Hsp60 construct, all plasmids exhibited robust *in vitro* stability across six consecutive passages, supporting their potential as reliable expression systems for heterologous antigens in therapeutic and vaccine applications.

**Conclusions:**

We developed five BioBrick™-compatible plasmids with distinct promoters for auxotrophic complementation and antigen expression in BCG ΔLeuD, eliminating the need for antibiotic-based selection. This standardized plasmid series reduces vector-related variability in comparative studies and provides a diverse promoter repertoire that may enhance the stability and efficacy of recombinant BCG ΔLeuD strains, representing a valuable advancement for prophylactic and therapeutic vaccine development.

**Supplementary Information:**

The online version contains supplementary material available at 10.1007/s11033-026-11830-x.

## Introduction

Synthetic biology aims to engineer biological systems capable of producing valuable biomolecules [1]. One of the most widely employed strategies involves the assembly of efficient expression cassettes for gene expression. However, traditional cloning techniques rely on the presence of restriction sites in plasmid vectors and are often unsuitable for constructing plasmids with multiple insertions or modular architectures [2]. The BioBrick™ standard, proposed by Tom Knight in 2003, introduced a physical cloning framework that enables the construction of standardized and modular DNA sequences [3]. Because synthetic biology frequently reuses functional elements—such as promoters, ribosome binding sites, tags, antibiotic resistance genes, and replication origins—BioBrick™ parts are designed to be interchangeable [4].This modularity facilitates the development of cloning systems, including integrative and episomal vectors, and enables greater optimization and automation, offering significant advantages over traditional molecular cloning approaches [3].

All BioBrick™-compatible DNA sequences are flanked by a characteristic arrangement of restriction sites recognized by type II restriction endonucleases [5]. The upstream region is flanked by *Eco*RI and *Xba*I sites, while the downstream region contains *Spe*I and *Pst*I sites. Upon ligation of two BioBrick™ parts, a short “scar” sequence is generated at the junction; this sequence is not recognized by any of the enzymes used in the assembly process [5, 6]. Importantly, ligation restores the *Eco*RI site and introduces a new *Xba*I site at the upstream end of the inserted DNA fragment. Similarly, the downstream end preserves the *Pst*I site and incorporates a new *Spe*I site [6]. This architecture enables the seamless and sequential assembly of multiple DNA parts while maintaining full compatibility with the BioBrick™ standard.

*Mycobacterium bovis* Bacillus Calmette-Guérin (BCG) is a live attenuated vaccine widely used for the prevention of tuberculosis [7]. However, its use presents several limitations, including reduced efficacy against adult pulmonary forms of the disease, safety concerns in immunocompromised individuals, and interference with tuberculosis diagnosis via tuberculin skin testing [8]. When BCG is used as a vaccine vector for the expression of heterologous antigens, selective pressure is typically achieved through the incorporation of antibiotic resistance genes. This approach is problematic, as antibiotic resistance markers are not maintained *in vivo* and raise concerns regarding biosafety, genetic stability, and vaccine efficacy. To address these challenges, auxotrophic BCG strains have been developed as a biosafe alternative that relies on complementation of essential metabolic genes [9]. In this strategy, the gene deleted from the bacterial genome is reintroduced on a plasmid, thereby restoring bacterial growth only in the presence of the plasmid. This provides stable in vivo selective pressure without the use of antibiotics, enhancing plasmid maintenance and recombinant strain stability [10].

In this context, the development of expression systems that are both free of antibiotic resistance markers and compatible with the BioBrick™ standard represents an efficient and promising strategy. Previous studies from our group have focused on the development of BioBrick™-compatible vectors [11], auxotrophic BCG strains [10], and recombinant vaccine platforms, with emphasis on their application in both prophylactic and therapeutic contexts [12, 13]. Therefore, the present study aims to develop and characterize new pUP500-derived BioBrick™ vectors tailored for the construction of leucine-auxotrophic recombinant BCG strains (rBCG ΔLeuD), with potential applications in future therapeutic and prophylactic strategies targeting diverse diseases.

## Materials and methods

### Bacterial strains and growth conditions

*Escherichia coli* strain TOP10 was cultured in Luria–Bertani (LB) medium at 37 °C, with kanamycin (Invitrogen) added to a final concentration of 25 µg/mL when required. *Mycobacterium bovis* BCG ΔLeuD [10] was grown at 37 °C in Middlebrook 7H9 broth (Difco) supplemented with 10% oleic acid–albumin–dextrose–catalase (OADC; Difco), 0.2% glycerol, and 0.05% Tween 80 (Sigma-Aldrich); or on Middlebrook 7H10 agar (Sigma-Aldrich) supplemented with 10% OADC and 0.2% glycerol. When necessary, L-leucine (Sigma-Aldrich) was added to a final concentration of 100 µg/mL.

### Reagents and DNA manipulation

The pUP500 vectors used in the present work were previously constructed by Oliveira et al. (2019) [11]. This plasmid series includes pUP500/PpAN, pUP500/P18kDa, pUP500/Pag85B, pUP500/Phsp60, and pUP500/PhspX, all of which share an identical backbone and differ only in the promoter region, enabling the modulation of heterologous gene expression. The expression cassette containing the *leuD* gene was amplified from pUP410, and the *egfp* sequence was amplified from pRSETEmGFP (Invitrogen), using primers described previously [11].

### Construction of auxotrophic complementation vectors based on pUP500 series according to Biobrick™ standard

The pAN-*leuD* expression cassette was amplified by PCR, digested with *Eco*RI and *Spe*I (New England Biolabs), and ligated upstream of the promoter region in pUP500 vectors previously digested with *Eco*RI and *Xba*I (New England Biolabs), using T4 DNA ligase (Invitrogen). The ligation products were then used to transform *E. coli* TOP10 electrocompetent cells by electroporation, and recombinant clones were selected on antibiotic-containing medium. Positive clones were confirmed by plasmid DNA extraction followed by restriction digestion with *Eco*RI and *Pst*I.

Next, the resulting plasmids containing the pAN-*leuD* cassette were digested with *Spe*I and *Pst*I. The *egfp* reporter gene, amplified by PCR and digested with *Xba*I and *Pst*I, was then inserted downstream of the promoter region into the pUP500/pAN-*leuD* constructs. The ligation products were again used to transform *E. coli* TOP10 cells by electroporation, and recombinant clones were confirmed by restriction digestion as previously described. To eliminate the kanamycin resistance gene, the constructs were digested with *Hind*III (ThermoFisher), which flanks the resistance marker. The resulting fragments were religated, and the final plasmids were used to transform electrocompetent BCG ΔLeuD cells. All BioBrick components were previously sequence-validated and were confirmed in this study by restriction profiling, PCR screening, and functional expression analysis.

### Transformation of BCG and analysis of eGFP expression

Electrocompetent BCG ΔLeuD cells were transformed with the plasmids *pUP500/pAN-leuD-PpAN: egfp*,* pUP500/pAN-leuD-P18kDa: egfp*,* pUP500/pAN-leuD-Pag85B: egfp*,* pUP500/pAN-leuD-Phsp60:egfp*,* and pUP500/pAN-leuD-PhspX: egfp.* Recombinant strains were selected on Middlebrook 7H10 agar lacking leucine. Transformed BCG strains were then cultured in selective 7H9 broth for five days until reaching an optical density (OD₆₀₀) equivalent to 1 × 10⁸ CFU/mL. Untransformed BCG ΔLeuD served as the negative control.

For qualitative fluorescence analysis, samples were imaged using a Leica TCS SP8 confocal laser scanning microscope (CLSM) at 200× magnification and 4× digital zoom, in duplicate. Sequential image acquisition was employed to prevent spectral overlap. eGFP fluorescence was visualized using a 488 nm laser, while DNA staining with 4′,6-diamidino-2-phenylindole (DAPI) was detected using a 405 nm Violet laser. The number of plasmid copies per bacterium was determined by quantitative real-time PCR (qPCR).

For fluorescence quantification, samples were stained with Hoechst 33,342 dye and subsequently analyzed using a CytoFLEX LX flow cytometer (Beckman Coulter). Bacterial cells were excited with a 488 nm Blue laser (eGFP) and 375 nm Near Ultra Violet laser (Hoechst 33342), and fluorescence emission was detected using a 525 nm filter (eGFP) and 450 nm filter (Hoechst 33342). The acquisition rate was set to 200 events s⁻¹, resulting in a total of 20,000 bacterial cells collected per sample. Non-bacterial events were excluded from the analysis based on forward and side scatter (FSC × SSC) profiles and Hoechst 33,342 negative fluorescence discrimination. The eGFP was analyzed for fluorescence discrimination (negative/positive) and fluorescence intensity. All analyses were conducted in duplicate.

### Functional analysis of pUP500 vectors in rBCG ΔLeuD strains grown inside macrophages

The J774.A1 murine macrophage cell line was cultured in DMEM supplemented with 10% fetal bovine serum under controlled conditions (37 °C, 5% CO₂, and 95% humidity) at a density of 1 × 10⁵ cells per well in 12-well plates (Corning). After 24 h, the cells were infected with recombinant BCG ΔLeuD (rBCG) at a multiplicity of infection (MOI) 1:10 (macrophage: BCG) and incubated for 3 h to allow phagocytosis. Non-internalized bacteria were removed by washing with 1× PBS, and the infected macrophages were maintained in culture for an additional 48 h.

In order to determine BCG internalization, a quantitative real-time PCR (qPCR) assay was performed. Total DNA from infected macrophages was extracted using the Bacteria Genomic Prep Kit (Cytiva), according to the manufacturer’s instructions. The primer pairs were designed to amplify the *sigH* gene for quantification of BCG DNA (forward: 5′-CCGACGCCGAGGACTTG-3′; reverse: 5′-CCGCTGTTTCTTGCGATAGC-3′) and the *β-actin* gene for quantification of macrophage DNA (forward: 5′-AGAGGGAAATCGTTGCGTGAC-3′; reverse: 5′-CAATAGTGATGACCTGGCCGT-3′) [14]. qPCR reactions were performed in triplicate using PowerUp™ SYBR™ Green Master Mix (Applied Biosystems, USA) in a LightCycler^®^ 96 system (Roche, Switzerland) under the following cycling conditions: an initial step at 50 °C for 2 min, followed by 95 °C for 2 min, and 40 cycles of 95 °C for 15 s and 60 °C for 1 min.

The expression of *eGFP* was assessed by confocal microscopy as previously described, with the addition of a 552 nm laser for Texas Red fluorescence detection, as well as by RT-qPCR. Briefly, infected macrophages were collected in TRI Reagent^®^ (Sigma-Aldrich, USA) and total RNA was extracted according to the manufacturer’s instructions. Complementary DNA (cDNA) was synthesized using the High-Capacity cDNA Reverse Transcription Kit (Applied Biosystems, USA). The qPCR reactions were performed using 100 ng of cDNA and the same cycling conditions described above. Relative gene expression was calculated using the 2^−ΔΔCt^ method, employing *β-actin* gene as the endogenous control and untransformed BCG ΔLeuD samples as calibrator. The *leuD* expression was also verified using the same parameters and the following primers: forward: 5′-TCTTTCTGAAGCGGGTCACC-3′; reverse: 5′-GCTTCCACAGGAGTTCCACA-3′.

### *In vitro* stability analysis

Strains of BCG ΔLeuD transformed with the recombinant plasmids were grown in Middlebrook 7H9 broth with or without L-leucine supplementation. The transformed strains were serially passaged six times by transferring 125 µL of stationary-phase culture into 4 mL of fresh medium every seven days. At each passage, an aliquot was collected, serially diluted, and plated onto Middlebrook 7H10 agar, again with or without L-leucine. Plates were incubated at 37 °C, and colonies were counted to evaluate plasmid retention. Plasmid stability was expressed as the proportion of plasmid-bearing colonies relative to the total colony count. Plasmid copy number per cell was determined using plasmid DNA extracted from bacterial cultures at the first and sixth generations, following the protocol described by Madiraju et al. (2000), with minor modifications. DNA concentration was measured in triplicate using a NanoDrop™ spectrophotometer (Thermo Fisher Scientific, USA). Plasmid copy number was calculated according to the following formula:

$$\begin{aligned} &\:\mathrm{P}\mathrm{l}\mathrm{a}\mathrm{s}\mathrm{m}\mathrm{i}\mathrm{d}\:\mathrm{C}\mathrm{o}\mathrm{p}\mathrm{y}\:\mathrm{N}\mathrm{u}\mathrm{m}\mathrm{b}\mathrm{e}\mathrm{r}\\&=\frac{\mathrm{D}\mathrm{N}\mathrm{A}\:\mathrm{c}\mathrm{o}\mathrm{n}\mathrm{c}\mathrm{e}\mathrm{n}\mathrm{t}\mathrm{r}\mathrm{a}\mathrm{t}\mathrm{i}\mathrm{o}\mathrm{n}\:\mathrm{X}\:\mathrm{A}\mathrm{v}\mathrm{o}\mathrm{g}\mathrm{a}\mathrm{d}\mathrm{r}{\mathrm{o}}^{{\prime\:}}\mathrm{s}\:\mathrm{c}\mathrm{o}\mathrm{n}\mathrm{s}\mathrm{t}\mathrm{a}\mathrm{n}\mathrm{t}}{\mathrm{p}\mathrm{l}\mathrm{a}\mathrm{s}\mathrm{m}\mathrm{i}\mathrm{d}\:\mathrm{l}\mathrm{e}\mathrm{n}\mathrm{g}\mathrm{t}\mathrm{h}\:\mathrm{X}\:650} \end{aligned}$$

where 650 corresponds to the average molecular weight (Da) of one base pair. The results were adjusted per bacterial cell considering optical density of cultures.

### Statistical analyses

Statistical analysis was performed using GraphPad prism 8.0.1 software. Percentage of fluorescent cells and fluorescence levels were analyzed using one-way analysis of variance (ANOVA), followed by Tukey’s post hoc test for multiple comparisons. Statistical significance was defined as *P* ≤ 0.05. All data are presented as mean ± SEM.

## Results and discussion

### Construction of a new expression plasmid series for auxotrophic complementation in mycobacteria

Several auxotrophic BCG strains have been developed to enable heterologous antigen expression without the use of antibiotic resistance genes [9, 10, 15]. Borsuk et al. previously developed a leucine auxotrophic strain of BCG (BCG ΔLeuD), which exhibited improved in vitro and in vivo stability, and constructed the plasmid pUP410 to complement the deleted *leuD* gene. This plasmid allows the insertion of heterologous genes under the control of the pAN promoter [10]. To expand the repertoire of available vectors for antigen expression in mycobacteria and to standardize DNA assembly for this purpose, our group previously developed a set of BioBrick™-compatible plasmids designed for mycobacterial applications [11]. These vectors incorporate distinct promoters—including pAN, Ag85B, 18 kDa, Hsp60, and HspX—since promoter strength is known to influence both metabolic fitness and strain stability.

Considering the broad potential of leucine auxotrophic BCG strains, we constructed BioBrick™-compatible vectors containing the *pAN–leuD* expression cassette for auxotrophic complementation. These plasmids establish an antibiotic-free system for antigen expression in mycobacteria. Insertion of the *pAN–leuD* cassette into the pUP500 vectors was confirmed by the ability of transformants to grow in non-selective medium. Notably, upstream integration of *pAN–leuD* preserved the downstream cloning sites in each pUP500 construct, allowing the insertion of homologous or heterologous genes according to the BioBrick™ standard. The *egfp* gene was subsequently cloned downstream of the promoters and used as a reporter to assess the functionality of each plasmid for heterologous antigen expression via fluorescence detection. Successful removal of the kanamycin resistance gene was verified by 1% agarose gel electrophoresis (Supplementary Material, Fig. 4).

**Fig. 1:**
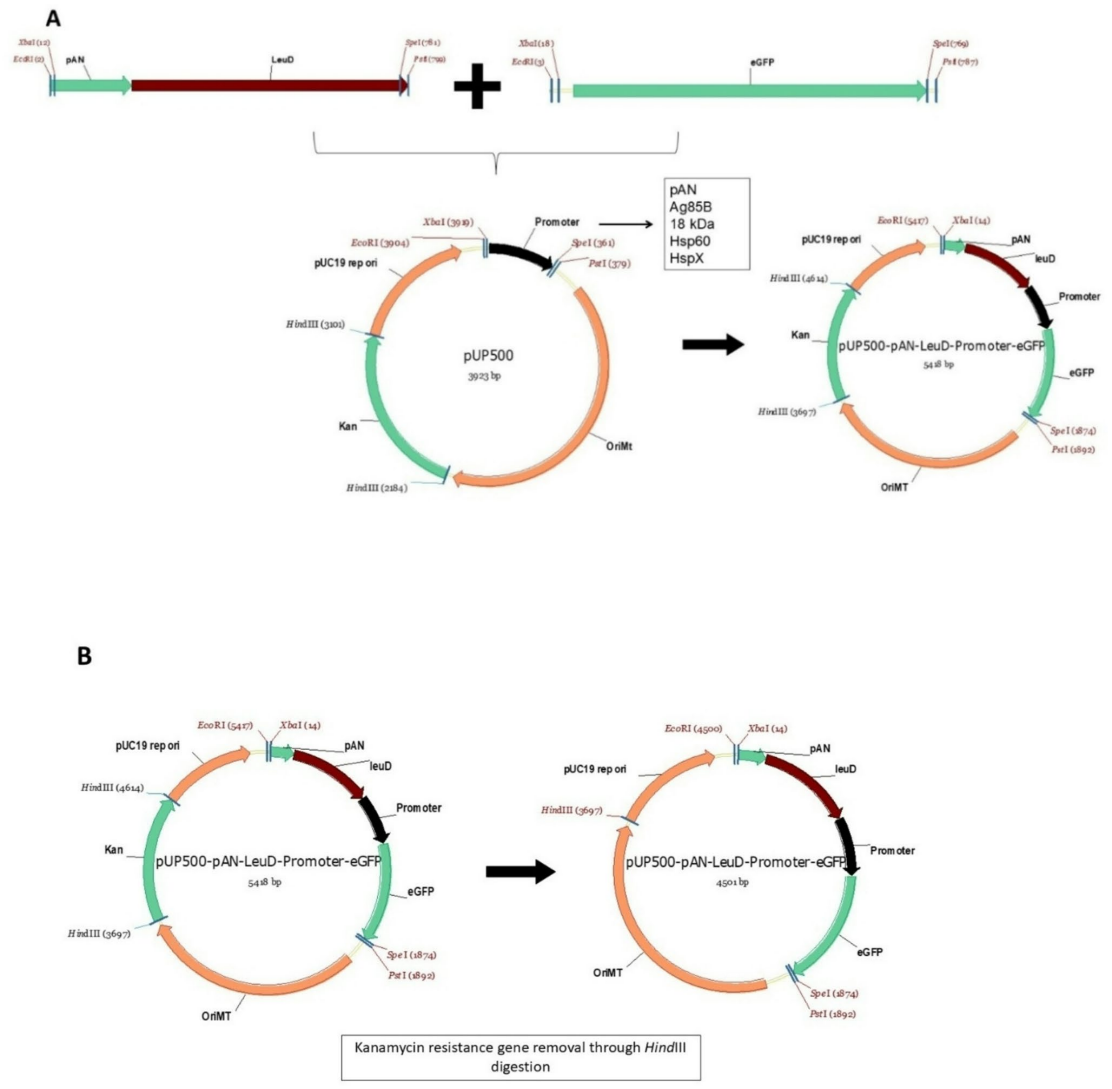
Schematic outline of the auxotrophic complementation vector construction. (**A**) The *pAN-leuD* cassette was retrieved from the pUP410 vector and inserted upstream of the promoter region in the pUP500 plasmids. The *eGFP* gene was subsequently cloned downstream of the promoter. (**B**) The kanamycin resistance gene was removed by digestion with the *Hind*III restriction enzyme

All plasmids generated in this study are listed in Table 1, and their structural organization is shown in Fig. 1. Importantly, this plasmid series enables direct and specific comparison of promoter activity, as all constructs share an identical vector backbone, eliminating variability associated with vector architecture.

**Table 1:** Plasmids constructed in this study and their *in vitro* stability

Plasmid constructions	Description	^a^In vitro stability of plasmids (%)	^b^Number of copies 1st | 6th generations	
pUP500/pAN-*leuD*-PpAN:*egfp Δ*kan^r^	Biobrick™ compatible vector for auxotrophic complementation with reporter gene *egfp* and without Kan^r^	100%	24 ± 3^*^	11 ± 1^*^
pUP500/pAN-*leuD*-P18kDa:*egfp Δ*kan^r^	Biobrick™ compatible vector for auxotrophic complementation with reporter gene *egfp* and without Kan^r^	89%	24.66 ± 1.15^*^	10.33 ± 3.6^*^
pUP500/pAN-*leuD*-Pag85B:*egfp Δ*kan^r^	Biobrick™ compatible vector for auxotrophic complementation with reporter gene *egfp* and without Kan^r^	100%	17 ± 2.30	17 ± 3.21
pUP500/pAN-*leuD*-Phsp60:*egfp Δ*kan^r^	Biobrick™ compatible vector for auxotrophic complementation with reporter gene *egfp* and without Kan^r^	23%	16.33 ± 3.46	13.33 ± 1
pUP500/pAN-*leuD*-PhspX:*egfp Δ*kan^r^	Biobrick™ compatible vector for auxotrophic complementation with reporter gene *egfp* and without Kan^r^	90%	25.66 ± 3.78^*^	9 ± 4.04^*^

^a^Plasmid stability was expressed as the proportion of plasmid-bearing colonies relative to the total colony count after six *in vitro* passages. ^b^Number of copies calculated for first and six generations of rBCG strains. Data are expressed as mean ± SD (*n* = 3) and asterisks represent statistical differences (*P* < 0.05) between generations of each strain

### Analyses of expression of eGFP in BCG ΔLeuD by confocal microscopy and flow cytometry

**Fig. 2:**
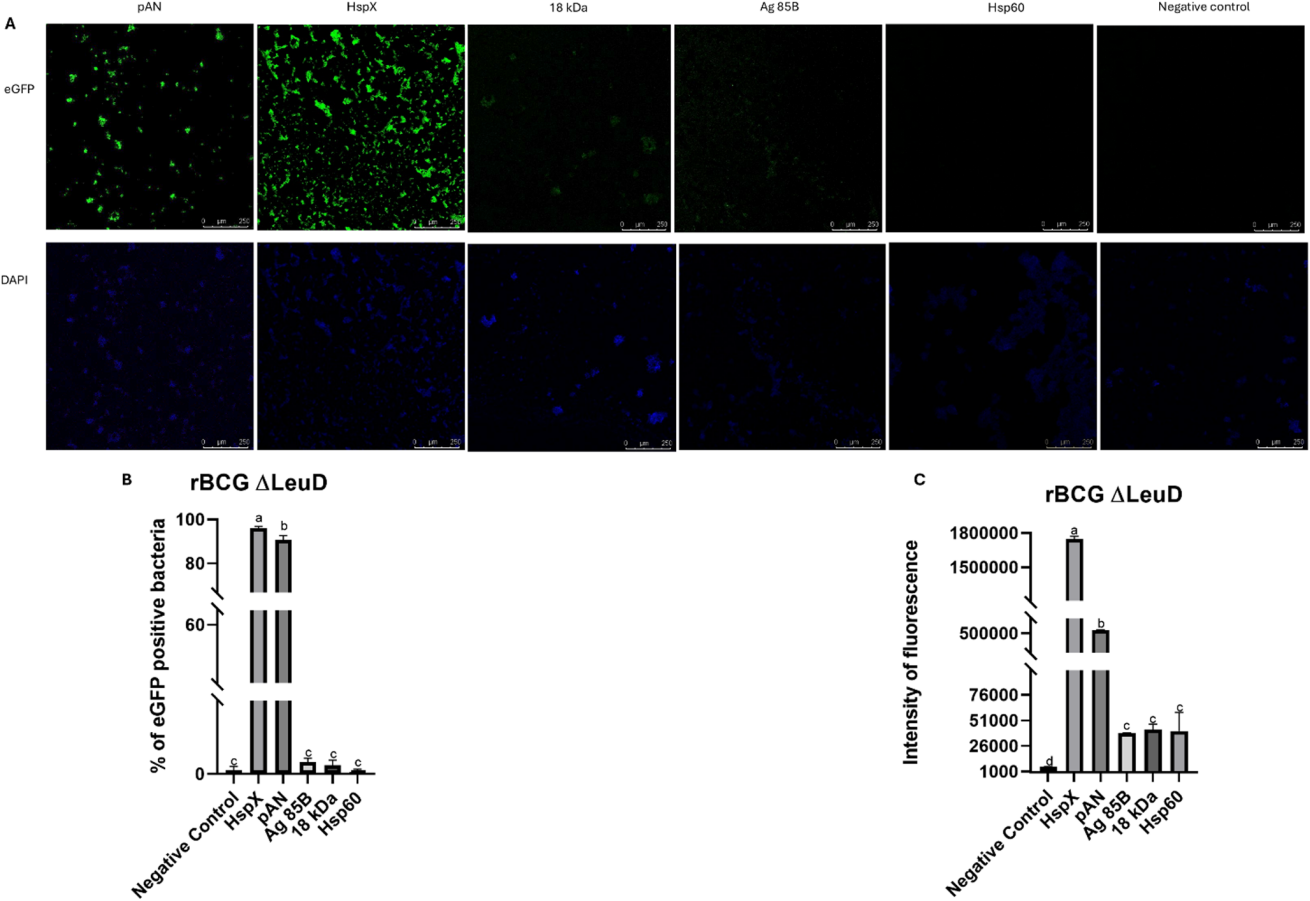
Fluorescence detection by confocal microscopy and flow cytometry in BCG ΔLeuD strains transformed with auxotrophic complementation plasmids. (**A**) Confocal microscopy of the recombinant cells. Individual fluorescence channels are shown: DAPI (nuclei, blue) and eGFP (green). (**B**) Percentage of eGFP positive bacteria cells assessed by flow cytometry. (**C**) Quantification of fluorescence intensity measured in gated eGFP-positive BCG cells grown in liquid medium using flow cytometry. Statistical analysis was performed using one-way ANOVA, and distinct letters denote significant differences among samples (*P* < 0.05)

Functional characterization of the plasmids by eGFP expression analysis using confocal microscopy and flow cytometry is presented in Fig. 2. Strains carrying plasmids with the pAN and HspX promoters exhibited markedly increased fluorescence intensity compared with the non-transformed BCG strain (negative control). In qualitative confocal analysis, the Ag85B and 18 kDa promoters produced weaker fluorescence than pAN and HspX but still generated a detectable signal above the negative control (Fig. 2A). No fluorescence was observed in strains transformed with the plasmid containing the Hsp60 promoter.

Flow cytometry confirmed these observations. Strains expressing eGFP under the HspX and pAN promoters showed a significantly higher percentage of fluorescent cells (Fig. 2B) and increased fluorescence intensity (*P* < 0.05) (Fig. 2C) relative to the control. Although strains expressing eGFP under the 18 kDa, Hsp60, and Ag85B promoters did not differ from the control in the proportion of fluorescent cells (*P* > 0.05), fluorescence intensity analysis revealed a significant increase in eGFP expression driven by these promoters (*P* < 0.05) (Fig. 2C).

The strong eGFP expression driven by the pAN promoter can be attributed to its high transcriptional stability, as previously demonstrated [16]. Similarly, the HspX promoter also supported high levels of eGFP expression in BCG ΔLeuD. Its stability has been reported both *in vitro *and within macrophages infected with avirulent strains of *M. tuberculosis* [17]. Additionally, HspX is known to respond to environmental stimuli, being inducible under hypoxic conditions and in the presence of nitric oxide and carbon monoxide, while maintaining expression stability [18].

The classical 18 kDa and Ag85B promoters did not induce strong eGFP expression *in vitro.* A recent study reported no significant GFP expression under the control of the Ag85B promoter in the pLA71 plasmid, attributing this finding to a negative influence of the plasmid backbone [19]. As for the 18 kDa promoter, it is known to be positively regulated in antigen-presenting cells, such as macrophages; however, its activity in liquid culture is generally weak, as previously demonstrated [20].

We observe a slight eGFP fluorescence under the control of the Hsp60 promoter, which is consistent with findings from previous studies [11, 21]. Earlier studies also support this observation: Zarouni et al. demonstrated that the Hsp60 promoter is unstable during or shortly after transformation, leading to impaired expression of heterologous genes in BCG [22]. This was further corroborated by Joseph et al., who reported partial deletions in the DNA sequence encoding the HIV gp120 protein when driven by the Hsp60 promoter in the pMV261 plasmid [23]. These findings likely explain the lack of detectable fluorescence observed in our experiments.

Collectively, these findings identify the pAN and HspX promoters as robust candidates for driving heterologous antigen expression in auxotrophic BCG strains, supporting consistent and elevated antigen production essential for effective immune activation. Although the 18 kDa promoter showed limited fluorescence *in vitro*, its inducible activation within antigen-presenting cells suggests a context-dependent advantage that may enhance intracellular antigen expression. In contrast, the Ag85B promoter demonstrated comparatively low transcriptional activity under the tested conditions, which should be considered when selecting regulatory elements, as insufficient antigen expression may compromise the strength of the immune response.

### Functionality analysis of vectors in macrophages

**Fig. 3:**
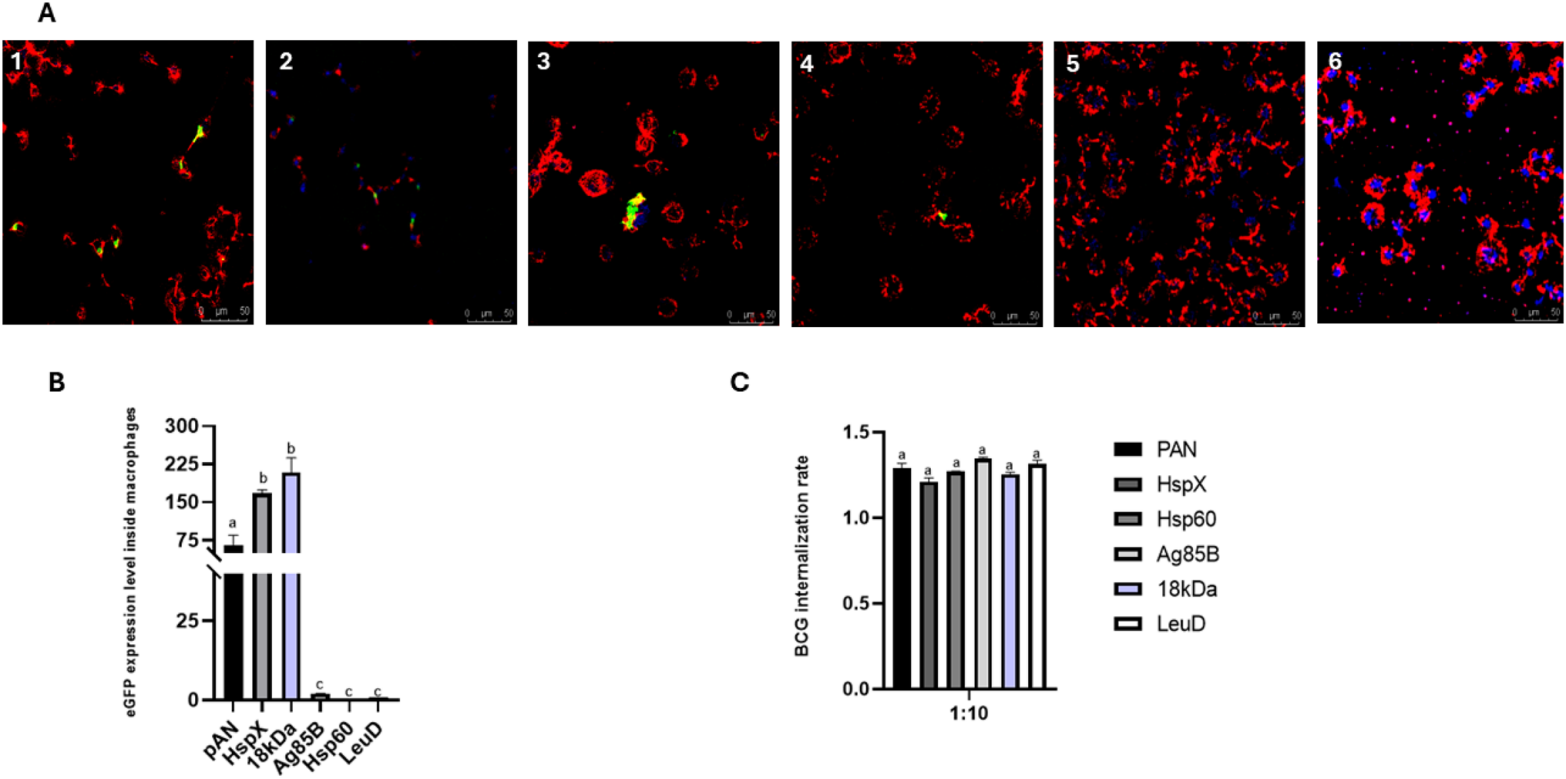
Analysis of *eGFP* expression levels and determination of the bacterial internalization rate in infected macrophages. (**A**) Fluorescence detection by confocal microscopy in macrophages infected with BCG ΔLeuD strains harboring auxotrophic complementation plasmids. *eGFP* expression is shown under the control of (1) pAN, (2) HspX, (3) 18 kDa, (4) Ag85B and (5) Hsp60 promoters. Panel (6) represents the non-transformed BCG ΔLeuD strain (negative control). Images were acquired at 200× magnification and 2× digital zoom. Merged images display the overlay of individual fluorescence channels: DAPI (nuclei, blue), eGFP (green), and Texas Red (cytoskeleton, red). (**B**) Quantification of *eGFP* expression levels inside macrophages using qPCR. (**C**) Determination of BCG internalization rate by macrophages at a MOI of 1:10 by qPCR. *eGFP* expression was calculated using the 2^−ΔΔCt^ method, with *β-actin* as the endogenous gene, and internalization rate was determined based on the ratio between *sigH* and *β-actin* copy numbers. Statistical analysis was performed using one-way ANOVA, and distinct letters denote significant differences among samples (*P* < 0.05)

Following the *in vitro* evaluation of eGFP fluorescence in recombinant BCG ΔLeuD strains, macrophages from the J774.A1 cell line were infected with the same strains and analyzed by confocal laser scanning microscopy (CLSM) and RT-qPCR. The strongest fluorescence signals were observed in rBCG strains expressing eGFP under the control of the pAN, HspX and 18 kDa promoters (Fig. 3A-B). No fluorescence was observed in macrophages infected with the BCG wild-type strain. Cytoskeletal labeling with Texas Red enabled clear visualization of cell boundaries and confirmed that the eGFP fluorescence was localized intracellularly (Fig. 3A).

Moreover, no detectable *eGFP* expression was observed inside macrophages under the control of the Hsp60 and Ag85B promoters, despite the BCG internalization (Fig. 3C) and *leuD* expression (data presented but not shown) having occurred at the same rate (*P* > 0.05) as the other strains. These results demonstrate that auxotrophic complementation occurred properly and was sufficient to ensure plasmid maintenance and viability within the cells, and lack of *eGFP* expression is probably attributed to individual promoter strength in the conditions evaluated. Notably, an increase in *eGFP* expression was observed under the control of the 18 kDa promoter in the intracellular environment. This result suggests promoter activation upon entry into host cells, consistent with previous observations reported by Dellagostin et al. (1995) [20].

The inducible nature of this promoter may represent a strategic advantage over constitutive promoters, as continuous gene expression can impose a metabolic burden on the bacterial cell. Moreover, activation specifically within immune cells may optimize antigen production at the site of host–pathogen interaction while minimizing unnecessary energy expenditure under extracellular conditions [20, 24].

Collectively, these findings indicate that promoter activity is strongly influenced by the intracellular environment and suggest that inducible promoters, such as 18 kDa, may enhance antigen presentation efficiency. Such regulatory elements may therefore represent promising tools for the development of recombinant BCG-based vaccine strategies [8, 20, 24].

In the present study, we successfully constructed functional plasmids compatible with BioBrick™ technology for the auxotrophic complementation of the BCG ΔLeuD strain. These plasmids were shown to be functional both in vitro and within macrophages, supporting their potential use in therapeutic and vaccine development strategies.

### *In vitro* stability analysis

Antibiotic resistance genes do not maintain selective pressure *in vivo*, a limitation of particular concern for recombinant live strains intended for vaccination or immunotherapy [25]. The absence of such pressure can lead to plasmid loss or reduced antigen expression, ultimately compromising strain stability and long-term vaccine efficacy [25, 26]. Auxotrophic strains therefore represent a promising antibiotic-free alternative for stable antigen expression. Additionally, promoter choice is well known to influence the stability of recombinant BCG strains, potentially affecting their therapeutic performance [10].

Promoter strength directly influences antigen expression, metabolic load, and overall immunogenicity [21]. Very strong promoters can boost antigen production but also increase metabolic burden, which may reduce cell fitness and promote plasmid loss or instability, ultimately leading to decreased expression over time. *In vivo*, these effects can alter antigen availability and quality, impacting the magnitude and consistency of the immune response. Thus, promoter strength must be balanced to avoid metabolic stress while ensuring stable plasmid maintenance and adequate immunogenicity [19, 21, 27].

In this context, our work addresses these challenges by providing a repertoire of *leuD*-based BCG vectors that share an identical molecular backbone and differ exclusively in their promoters. This standardized architecture enables modular, predictable cloning of heterologous genes and allows comparative analyses in which observed differences can be attributed solely to promoter-driven effects. Table 1 further presents the stability profile of the constructed plasmids and the number of copies along six generations. All plasmids—except that with the Hsp60 promoter—were consistently maintained, supporting sustained bacterial growth under selective pressure and indicating stable plasmid retention. These findings suggest that auxotrophic complementation effectively contributes to plasmid maintenance without the need for antibiotic selection, reinforcing the stability of the recombinant strains. The low plasmid copy number across generations offer advantages related to reduced metabolic burden, better growth rates and reduced protein-associated toxicity. These results support the suitability of this system for applications requiring stable heterologous gene expression over multiple bacterial generations. This feature is particularly relevant for therapeutic applications, as plasmid loss or antigen silencing often results in weakened or ineffective immune responses [26]. The Hsp60 plasmid exhibited lower stability compared with the other constructs, in agreement with the fluorescence analyses and with data reported in previous studies [22]. Overall, the plasmids developed in this study demonstrated robust *in vitro *stability, reinforcing their potential as reliable expression systems for heterologous antigens in both therapeutic and vaccine contexts.

## Conclusion

In conclusion, this study establishes a unified and standardized modular plasmid platform in *Mycobacterium bovis* BCG ΔLeuD that integrates BioBrick-derived backbones, previously characterized mycobacterial promoters, and auxotrophic complementation into a directly comparable system. By enabling controlled evaluation of promoter strength under identical experimental conditions, this platform reduces inter-study variability and supports rational promoter selection. The substitution of antibiotic resistance markers with auxotrophic complementation addresses important biosafety and regulatory concerns, minimizing the theoretical risk of horizontal gene transfer and improving alignment with current expectations for recombinant live bacterial vaccines.

Quantitative *in vitro* analyses, including real-time PCR and flow cytometry, confirmed consistent transcriptional and protein-level expression across constructs, both in culture and within macrophages, especially those employing pAN, HspX and 18 kDa promoters. Furthermore, stable plasmid maintenance under auxotrophic selection underscore the robustness and practical applicability of this system. Collectively, these findings provide a reliable and biosafety-oriented framework for the rational design and optimization of recombinant ΔLeuD BCG-based vaccine platforms.

## Electronic Supplementary Material

Below is the link to the electronic supplementary material.

Supplementary Material 1

## Data Availability

No datasets were generated or analysed during the current study.

## References

[1] Armianinova DK, Karpov DS, Kotliarova MS, Goncharenko AV (2022) Genetic Engineering in *Mycobacteria*. Mol Biol 56:830–841. 10.1134/S0026893322060036 10.31857/S0026898422060040 36475477

[2] Wang T, Ma X, Zhu H, Li A, Du G, Chen J (2012) Available methods for assembling expression cassettes for synthetic biology. Appl Microbiol Biotechnol 93:1853–1863. 10.1007/s00253-012-3920-8 22311648 10.1007/s00253-012-3920-8

[3] Shetty RP, Endy D, Knight TF (2008) Engineering BioBrick vectors from BioBrick parts. J Biol Eng 2:5. 10.1186/1754-1611-2-5 18410688 10.1186/1754-1611-2-5 PMC2373286

[4] Vick JE, Johnson ET, Choudhary S, Bloch SE, Lopez-Gallego F, Srivastava P, Tikh IB, Wawrzyn GT, Schmidt-Dannert C (2011) Optimized compatible set of BioBrick^™^ vectors for metabolic pathway engineering. Appl Microbiol Biotechnol 92:1275–1286. 10.1007/s00253-011-3633-4 22033566 10.1007/s00253-011-3633-4

[5] Matsumura I (2020) Methylase-assisted subcloning for high throughput BioBrick assembly. PeerJ 8:e9841. 10.7717/peerj.9841 32974095 10.7717/peerj.9841 PMC7489255

[6] Chao R, Yuan Y, Zhao H (2015) Recent advances in DNA assembly technologies. FEMS Yeast Res 15:1–9. 10.1111/1567-1364.12171 24903193 10.1111/1567-1364.12171 PMC4257898

[7] Matsuo K, Yasutomi Y (2011) *Mycobacterium bovis* bacille calmette-guérin as a vaccine vector for global infectious disease control. Tuberc Res Treat 2011:574591. 10.1155/2011/574591 22567267 10.1155/2011/574591 PMC3335490

[8] Bastos RG, Borsuk S, Seixas FK, Dellagostin OA (2009) Recombinant *Mycobacterium bovis* BCG. Vaccine 27:6495–6503. 10.1016/j.vaccine.2009.08.044 19720367 10.1016/j.vaccine.2009.08.044

[9] Dellagostin OA, Borsuk S, Oliveira TL, Seixas FK, Dellagostin OA, Borsuk S, Oliveira TL, Seixas FK (2022) Auxotrophic *Mycobacterium bovis* BCG: Updates and perspectives. Vaccines 10. 10.3390/vaccines10050802 10.3390/vaccines10050802 PMC9146772 35632558

[10] Borsuk S, Mendum TA, Fagundes MQ, Michelon M, Cunha CW, McFadden J, Dellagostin OA (2007) Auxotrophic complementation as a selectable marker for stable expression of foreign antigens in *Mycobacterium bovis* BCG. Tuberculosis 87:474–480. 10.1016/j.tube.2007.07.006 17888740 10.1016/j.tube.2007.07.006

[11] Oliveira TL, Stedman A, Rizzi C, Dorneles J, da Cunha CEP, Junior ASV, Dellagostin OA, McFadden J (2019) A standardized BioBrick toolbox for the assembly of sequences *in mycobacteria*. Tuberculosis 119:101851. 10.1016/j.tube.2019.07.002 31563455 10.1016/j.tube.2019.07.002

[12] Rizzi C, Bianco MV, Blanco FC, Soria M, Gravisaco MJ, Montenegro V, Vagnoni L, Buddle B, Garbaccio S, Delgado F, Leal KS, Cataldi AA, Dellagostin OA, Bigi F (2012) Vaccination with a BCG Strain Overexpressing Ag85B Protects Cattle against *Mycobacterium bovis* Challenge. PLoS ONE 7:e51396. 10.1371/journal.pone.0051396 23251517 10.1371/journal.pone.0051396 PMC3519572

[13] Begnini KR, Rizzi C, Campos VF, Borsuk S, Schultze E, Yurgel VC, Nedel F, Dellagostin OA, Collares T, Seixas FK (2013) Auxotrophic recombinant *Mycobacterium bovis* BCG overexpressing Ag85B enhances cytotoxicity on superficial bladder cancer cells *in vitro*. Appl Microbiol Biotechnol 97:1543–1552. 10.1007/s00253-012-4416-2 23053076 10.1007/s00253-012-4416-2

[14] Park ST, Kang C-M, Husson RN (2008) Regulation of the *SigH* stress response regulon by an essential protein kinase in *Mycobacterium tuberculosis*. Proc Natl Acad Sci 105:13105–13110. 10.1073/pnas.0801143105 18728196 10.1073/pnas.0801143105 PMC2529121

[15] Moraes L, Trentini MM, Fousteris D, Eto SF, Chudzinski-Tavassi AM, Leite LC, de Kanno C AI (2022) CRISPR/Cas9 approach to generate an auxotrophic BCG strain for unmarked expression of LTAK63 adjuvant: a tuberculosis vaccine candidate. Front Immunol 13:867195. 10.3389/fimmu.2022.867195 35432328 10.3389/fimmu.2022.867195 PMC9005855

[16] Medeiros MA, Dellagostin OA, Armôa GRG, Degrave WM, de Mendonça-Lima L, Lopes MQ, Costa JF, Mcfadden J, McIntosh D (2002) Comparative evaluation of *Mycobacterium vaccae* as a surrogate cloning host for use in the study of mycobacterial genetics. Microbiology 148:1999–2009. 10.1099/00221287-148-7-1999 12101288 10.1099/00221287-148-7-1999

[17] Dokladda K, Billamas P, Palittapongarnpim P (2015) Different behaviours of promoters in *Mycobacterium tuberculosis* H37Rv and H37Ra. World J Microbiol Biotechnol 31:407–413. 10.1007/s11274-014-1794-x 25556328 10.1007/s11274-014-1794-x

[18] Abramovitch RB (2018) *Mycobacterium tuberculosis* reporter strains as tools for drug discovery and development. IUBMB Life 70:818–825. 10.1002/iub.1862 29707888 10.1002/iub.1862

[19] Nascimento LV, Santos CC, Leite LC, Nascimento IP (2020) Characterisation of alternative expression vectors for recombinant Bacillus Calmette-Guérin as live bacterial delivery systems. Mem Inst Oswaldo Cruz 115:e190347. 10.1590/0074-02760190347 32428188 10.1590/0074-02760190347 PMC7227789

[20] Dellagostin OA, Esposito G, Eales L-J, Dale JW, McFadden J (1995) Activity of mycobacterial promoters during intracellular and extracellular growth. Microbiology 141:1785–1792. 10.1099/13500872-141-8-1785 7551043 10.1099/13500872-141-8-1785

[21] Kanno AI, Goulart C, Rofatto HK, Oliveira SC, Leite LC, McFadden J (2016) New recombinant *Mycobacterium bovis* BCG expression vectors: improving genetic control over mycobacterial promoters. Appl Environ Microbiol 82:2240–2246. 10.1128/AEM.03677-15 26850295 10.1128/AEM.03677-15 PMC4959472

[22] Al-Zarouni M, Dale JW (2002) Expression of foreign genes in *Mycobacterium bovis* BCG strains using different promoters reveals instability of the *hsp60* promoter for expression of foreign genes in *Mycobacterium bovis* BCG strains. Tuberculosis 82:283–291. 10.1054/tube.2002.0374 12623271 10.1054/tube.2002.0374

[23] Joseph J, Fernández-Lloris R, Pezzat E, Saubi N, Cardona P-J, Mothe B, Gatell JM (2010) Molecular Characterization of heterologous HIV-1gp120 gene expression disruption in mycobacterium bo*vis* BCG host strain: a critical issue for engineering mycobacterial based-vaccine vectors. BioMed Res Int 2010:357370. 10.1155/2010/357370 10.1155/2010/357370 PMC2896670 20617151

[24] Chapman R, Chege G, Shephard E, Stutz H, Williamson A-L (2010) Recombinant *Mycobacterium bovis* BCG as an HIV vaccine vector. Curr HIV Res 8:282–298. 10.2174/157016210791208686 20353397 10.2174/157016210791208686 PMC3188323

[25] Mahant A, Saubi N, Eto Y, Guitart N, Gatell JM, Hanke T, Joseph J (2017) Preclinical development of BCG.HIVA^2auxo.int^, harboring an integrative expression vector, for a HIV-TB Pediatric vaccine. Enhancement of stability and specific HIV-1 T-cell immunity. Hum Vaccines Immunother 13:1798–1810. 10.1080/21645515.2017.1316911 10.1080/21645515.2017.1316911 PMC5557246 28426273

[26] Rizzi C, Peiter AC, Oliveira TL, Seixas ACP, Leal KS, Hartwig DD, Seixas FK, Borsuk S, Dellagostin OA (2017) Stable expression of *Mycobacterium bovis* antigen 85B in auxotrophic *M. bovis* bacillus Calmette-Guérin. Mem Inst Oswaldo Cruz 112:123–130. 10.1590/0074-02760160360 28177046 10.1590/0074-02760160360 PMC5293121

[27] Oliveira TL, Rizzi C, Dellagostin OA (2017) Recombinant BCG vaccines: molecular features and their influence in the expression of foreign genes. Appl Microbiol Biotechnol 101:6865–6877. 10.1007/s00253-017-8439-6 28779291 10.1007/s00253-017-8439-6

