# Supplementary material for: A versatile plasmid platform for auxotrophic complementation in attenuated Mycobacterium bovis BCG
Source: Mol Biol Rep. 2026 Apr 28;53(1):679. doi: 10.1007/s11033-026-11830-x (PMC13124767; doi:10.1007/s11033-026-11830-x)
Supplement: Supplementary file 1 — Supplementary Material 1 [file 11033_2026_11830_MOESM1_ESM.pdf]

Molecular Biology Reports

A Versatile Plasmid Platform for Auxotrophic Complementation in Attenuated *Mycobacterium bovis* BCG

Andriele Bonemann Madruga[a]; Jady Duarte Nogueira[a]; Mara Andrade Colares Maia[a]; Natasha Rodrigues de Oliveira[a]; Maria Eduarda Ehlert[b]; Valentina Gessinger Ferreira[b]; Bruna Silveira Pacheco[b]; Fabiana Kommling Seixas[b]; Tiago Veiras Collares[b]; Izani Bonel Acosta[c]; Antônio Sergio Varela Junior[c]; Odir Antônio Dellagostin[a]; Thaís Larré Oliveira Bohn[a #]

[a]Laboratório de Vacinologia, Centro de Desenvolvimento Tecnológico, Universidade Federal de Pelotas, Pelotas, Rio Grande do Sul, Brasil.

[b]Laboratório de Biotecnologia do Câncer, Centro de Desenvolvimento Tecnológico, Universidade Federal de Pelotas, Pelotas, Rio Grande do Sul, Brasil.

[c]Grupo de Pesquisa em Reprodução Animal Comparada, Faculdade de Medicina Veterinária, Universidade Federal de Pelotas, Pelotas, Rio Grande do Sul, Brasil.

[#]Corresponding author: thais.larre@ufpel.edu.br

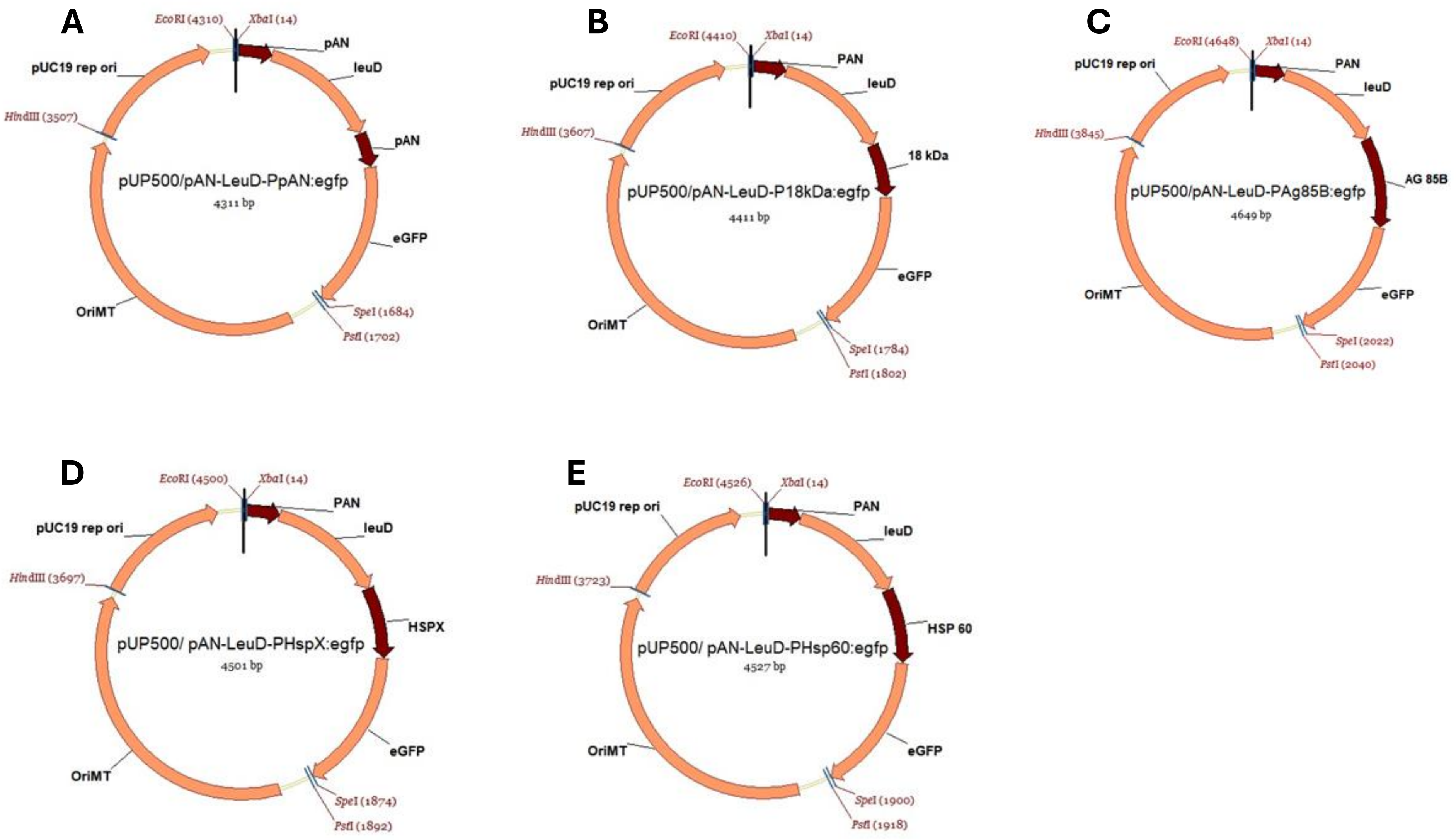

Figure 1: Schematic representation of Biobrick™ vectors constructed.

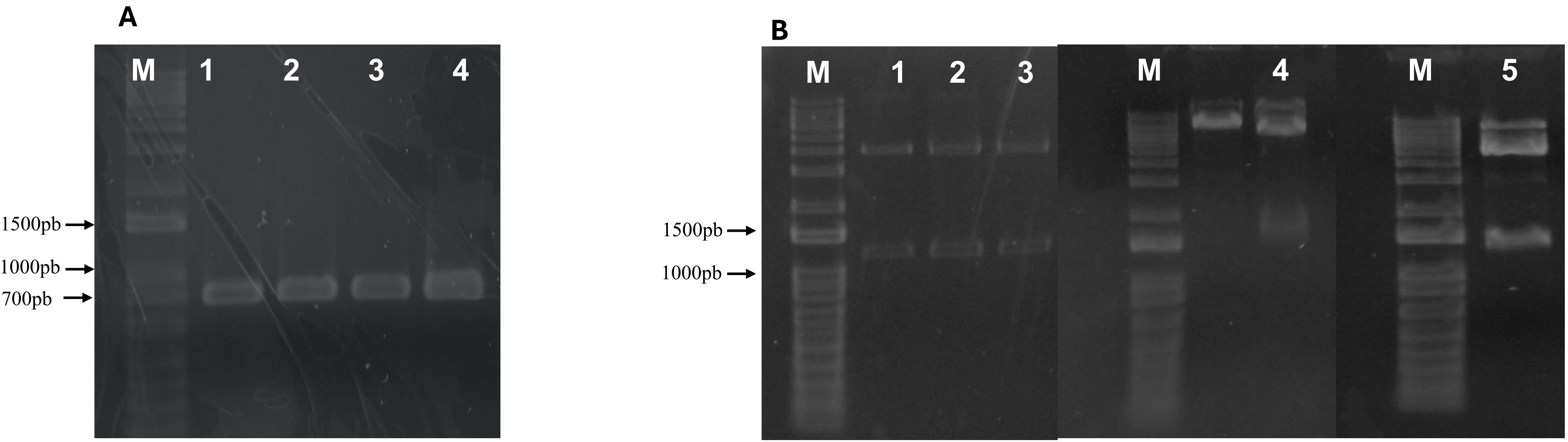

Figure 2. Cloning of the *pAN–LeuD* cassette into vectors of the pUP500 series. **(A)** Amplification of the *pAN–LeuD* cassette. M, 1 kb Plus DNA ladder; lanes 1–4, amplified *pAN–LeuD* cassette. **(B)** Restriction digestion to confirm insertion of the *pAN–LeuD* cassette into pUP500 series vectors. M, 1 kb Plus DNA ladder; lane 1, *pUP500–pAN–LeuD/18 kDa*; lane 2, *pUP500–pAN–LeuD/pAN*; lane 3, *pUP500–pAN–LeuD/HspX*; lane 4, *pUP500–pAN–LeuD/Hsp60*; lane 5, *pUP500–pAN–LeuD/Ag85B*. Digestion reactions were performed using *Eco*RI and *Spe*I. Notably, the released insert comprises the *pAN–LeuD* cassette plus the promoter sequence cloned in each vector.

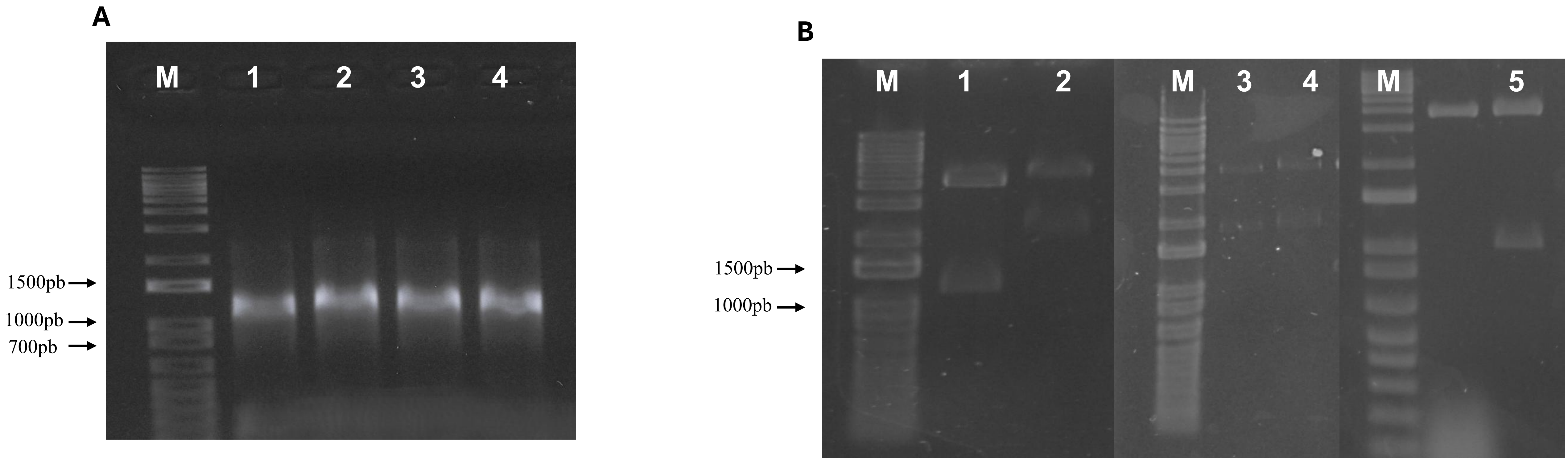

Figure 3. (A) Amplification of the *eGFP* coding sequence and confirmation of its insertion into the constructed vectors. M, 1 kb Plus DNA ladder; lanes 1–4, amplified *eGFP* fragment. **(B)** Restriction digestion analysis to confirm ligation of the eGFP insert into the vectors generated in the previous step. M, 1 kb Plus DNA ladder; lane 1, *pUP500–pAN–LeuD/pAN–eGFP*; lane 2, *pUP500–pAN–LeuD/Ag85B–eGFP*; lane 3, *pUP500–pAN–LeuD/Hsp60–eGFP*; lane 4, *pUP500–pAN–LeuD/HspX–eGFP*; lane 5, *pUP500–pAN–LeuD/18 kDa–eGFP*. Digestion reactions were performed using *Eco*RI and *Pst*I. Notably, the released insert comprises the *pAN–LeuD* cassette plus the promoter sequence cloned in each vector and *eGFP*.

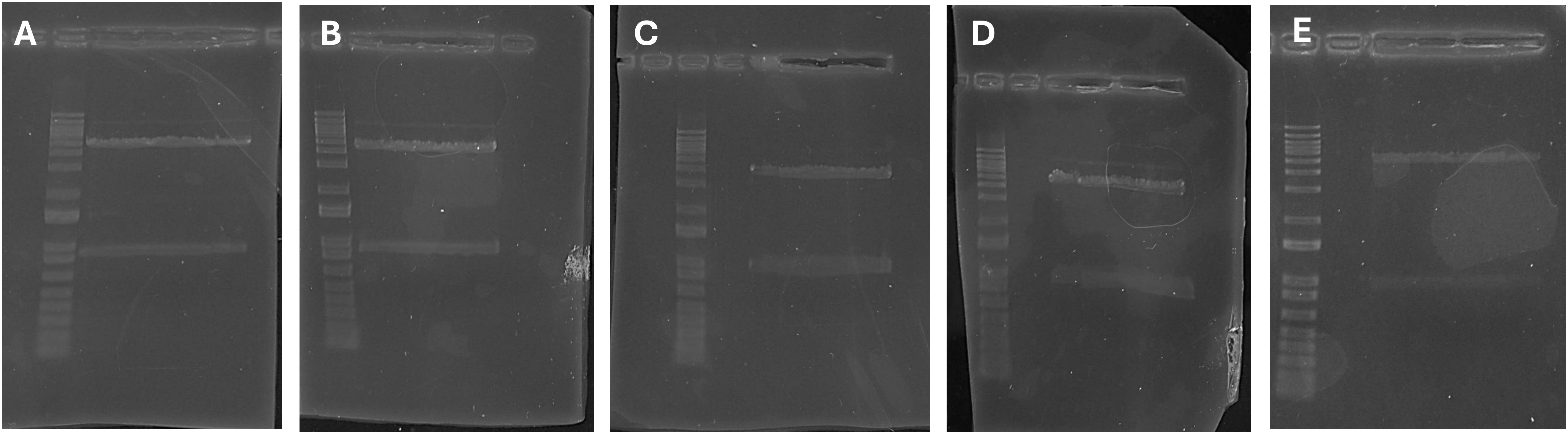

Figure 4: Agarose gel electrophoresis (1%) of kanamycin resistance gene removal (960pb) using the *Hind*III enzyme. **(A)** *pUP500/pAN-leuD-pHSP60:egfp Δkan^r* **(B)** *pUP500/pAN-leuD-PpAN:egfp Δkan^r* **(C)** *pUP500/pAN-leuD-pHspX:egfp Δkan^r* **(D)** *pUP500/pAN-leuD-P18kDa:egfp Δkan^r* **(E)** *pUP500/pAN-leuD-pAg85B:egfp Δkan^r*
